# Supplementary figures and images for: DNA segment capture by Smc5/6 holocomplexes
Source: Nat Struct Mol Biol. 2023 Apr 3;30(5):619–28. doi: 10.1038/s41594-023-00956-2 (PMC10191858; doi:10.1038/s41594-023-00956-2)

Fig. 1C

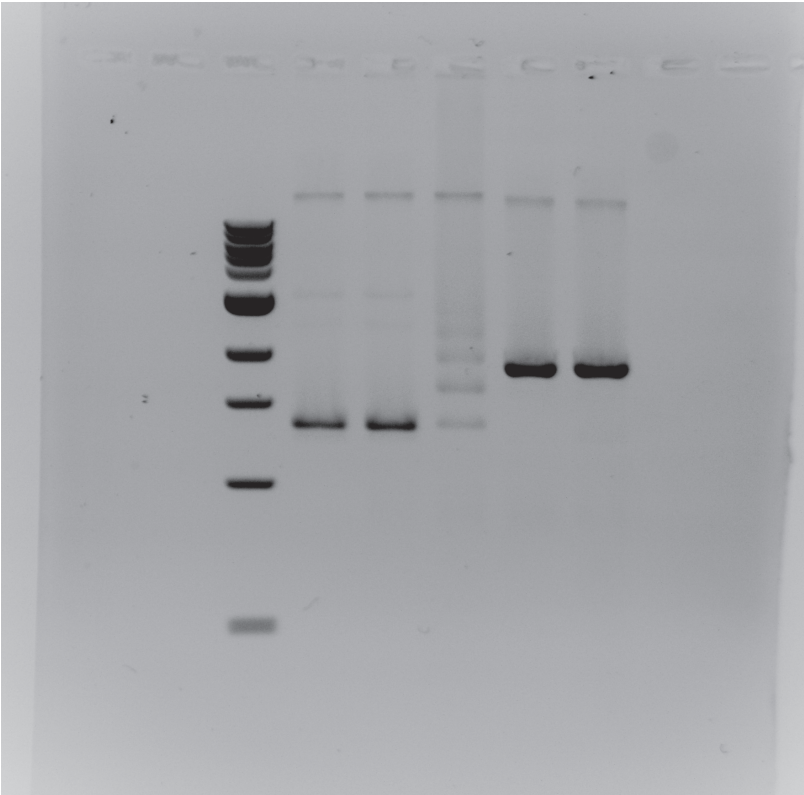

Fig. 1D

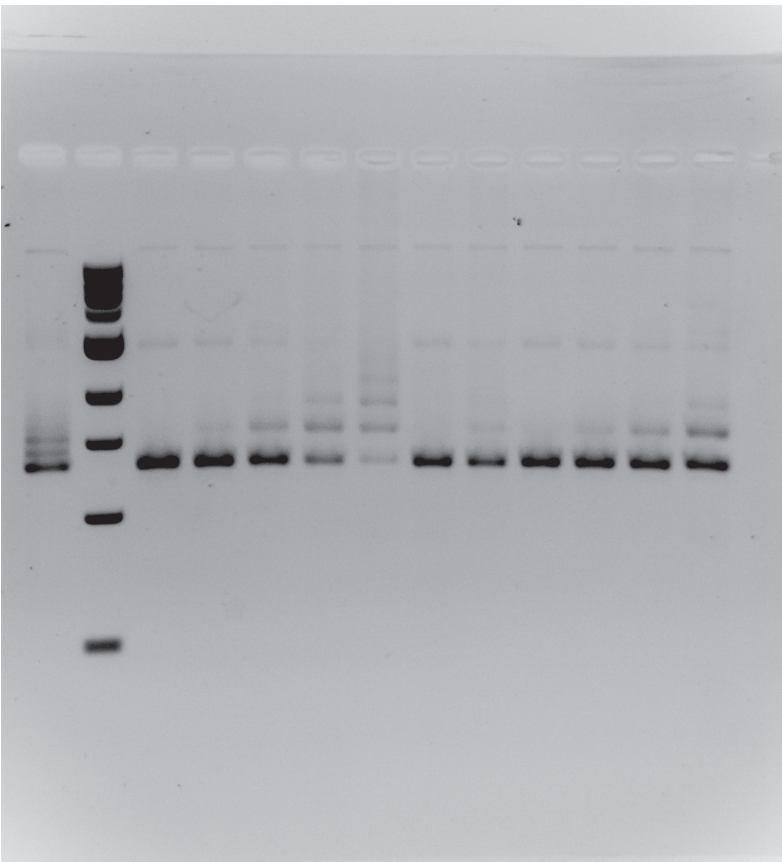

Supplement: Source Data Fig. 1 — Uncropped agarose gel pictures for Fig. 1. [file 41594_2023_956_MOESM4_ESM.pdf]

Fig. 2B

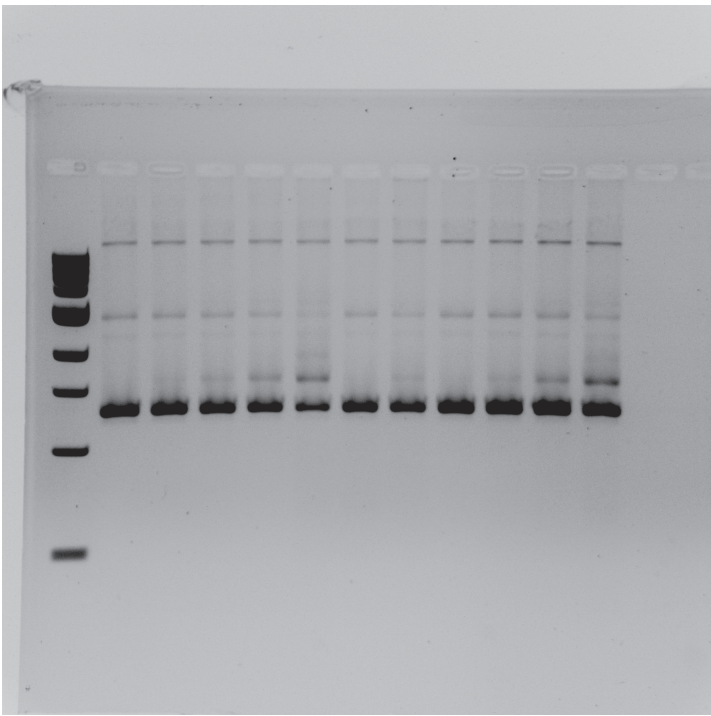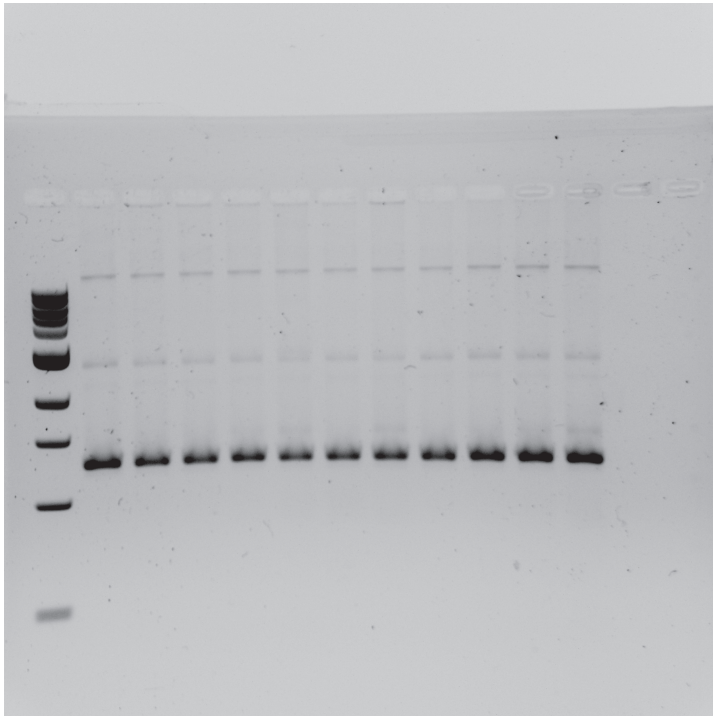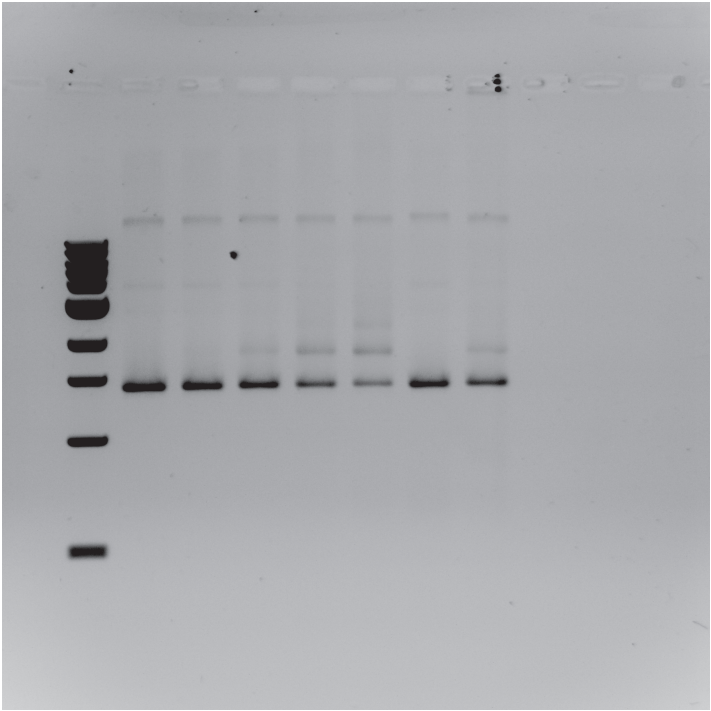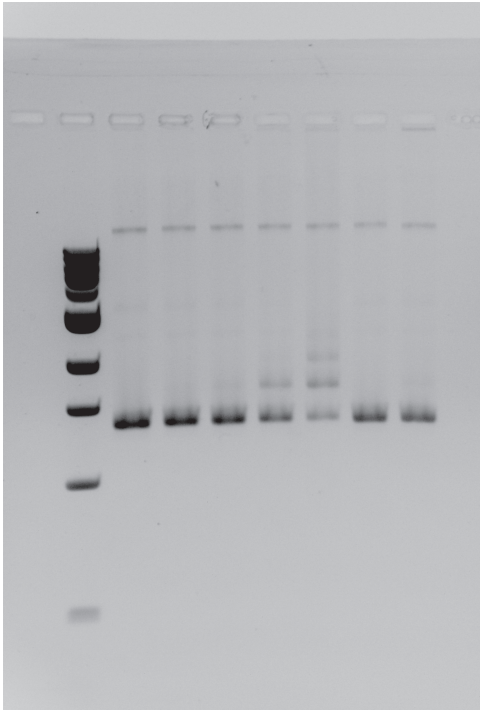

Supplement: Source Data Fig. 2 — Uncropped agarose gel pictures for Fig. 2. [file 41594_2023_956_MOESM5_ESM.pdf]

Fig. 3

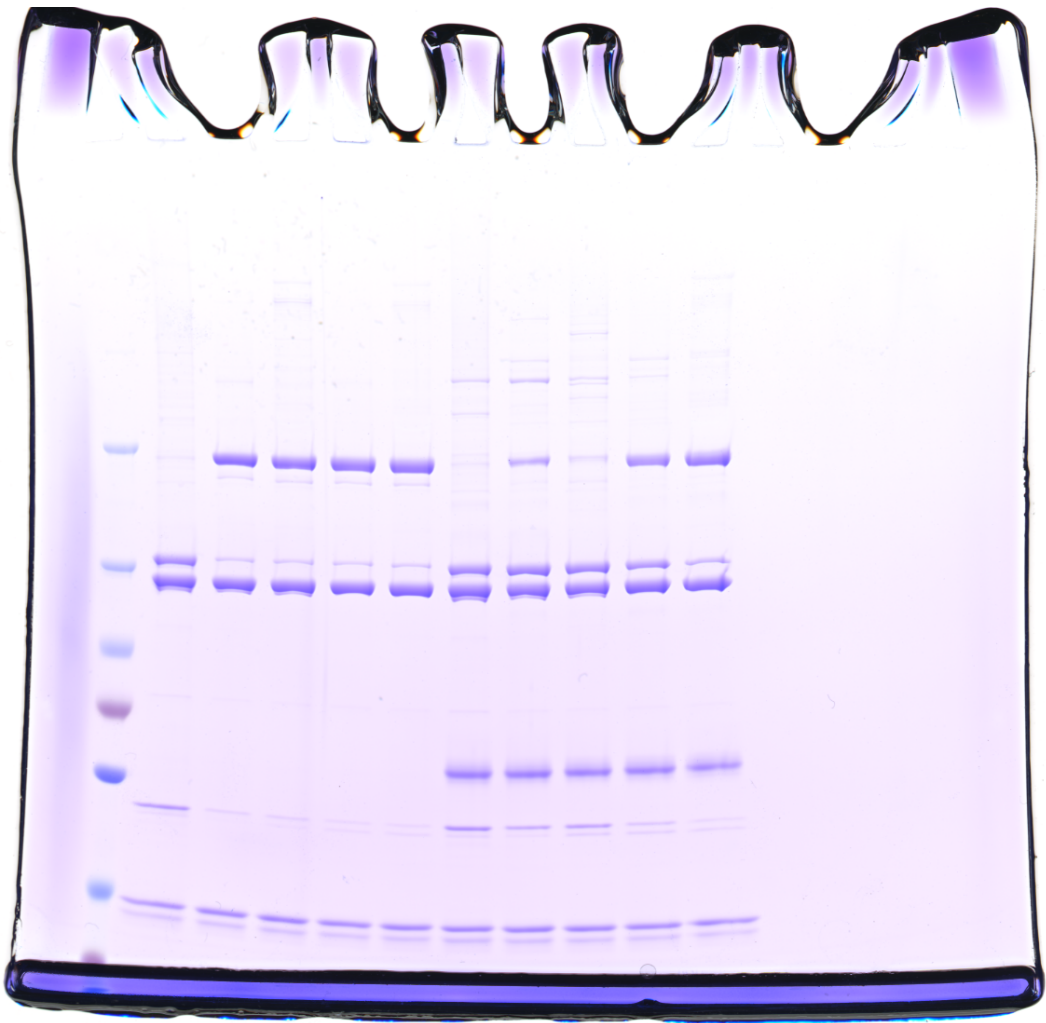

Supplement: Source Data Fig. 3 — Uncropped SDS–PAGE picture for Fig. 3. [file 41594_2023_956_MOESM6_ESM.pdf]

Fig. 4B and C

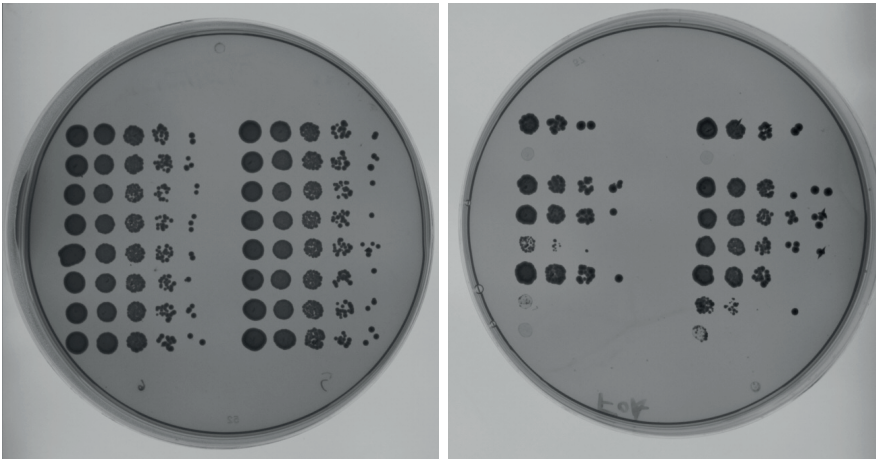

Fig. 4D

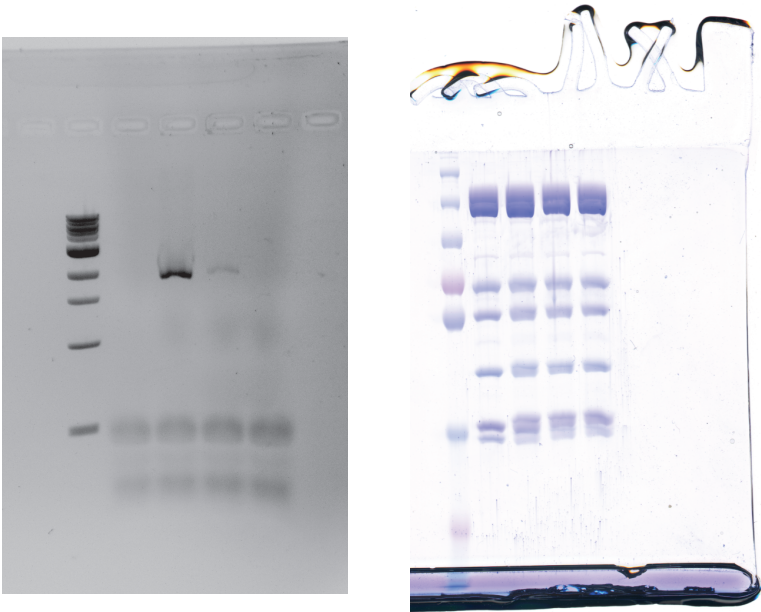

Fig. 4E

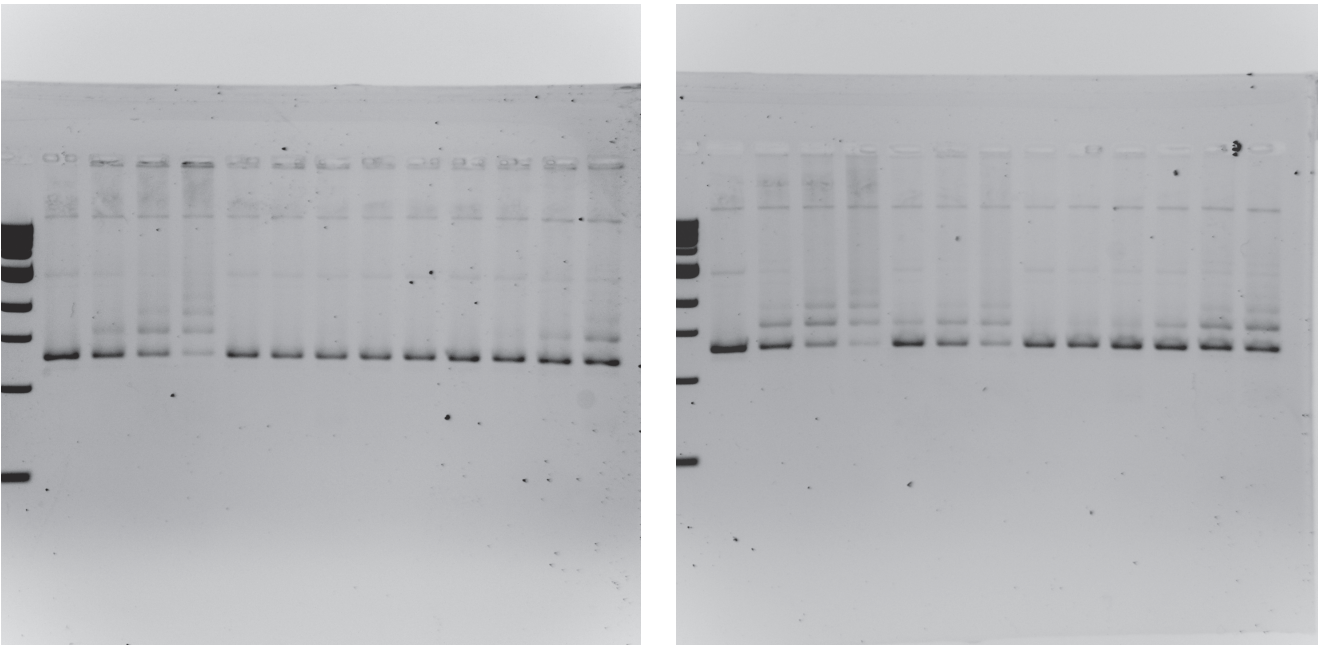

Supplement: Source Data Fig. 4 — Uncropped pictures of agarose gels, protein gels and yeast spotting plates for Fig. 4. [file 41594_2023_956_MOESM7_ESM.pdf]

Fig. 5A

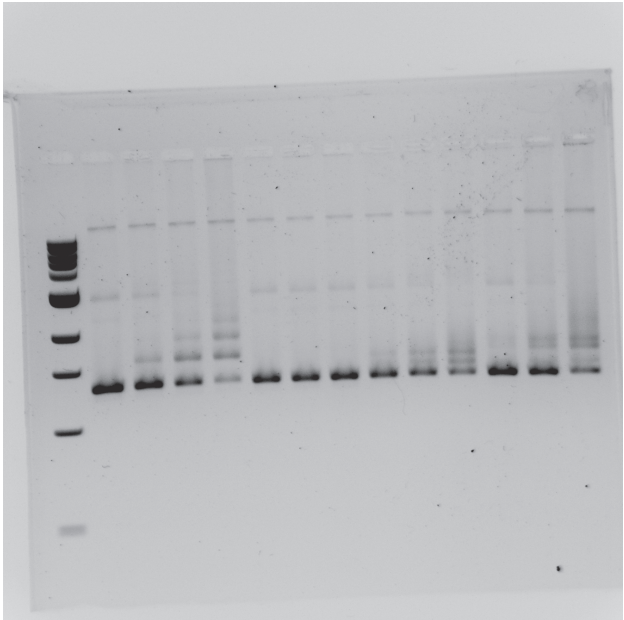

Fig. 5C

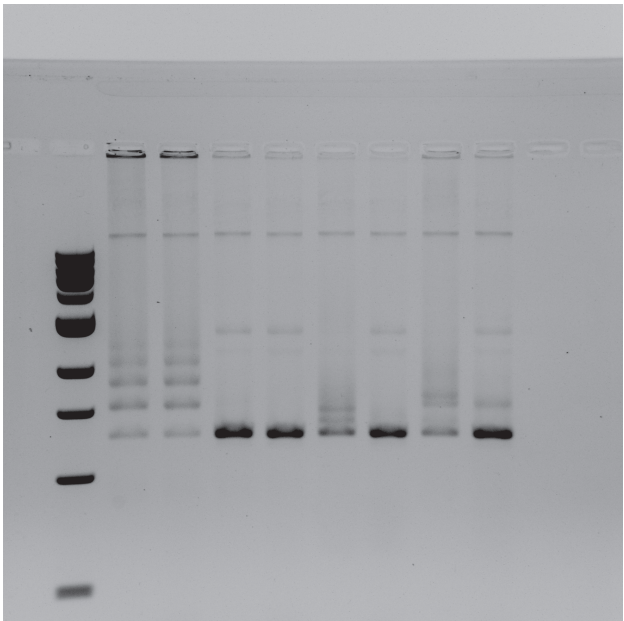

Fig. 5D

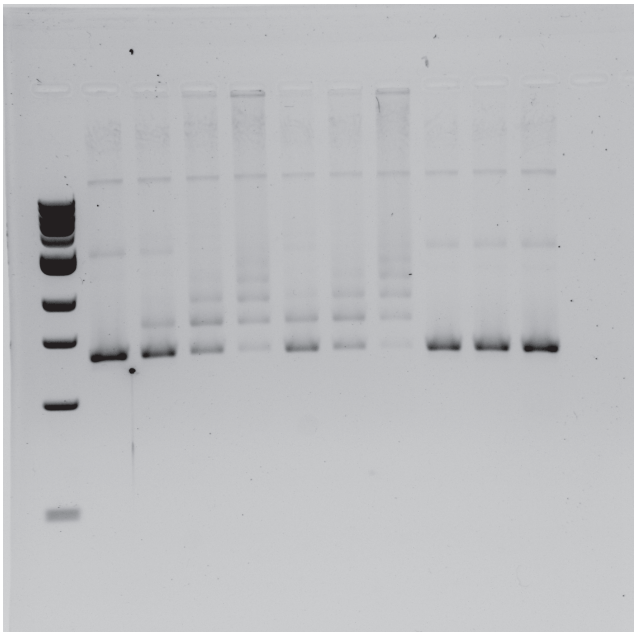

Supplement: Source Data Fig. 5 — Uncropped agarose gel pictures for Fig. 5. [file 41594_2023_956_MOESM8_ESM.pdf]

Fig. 6

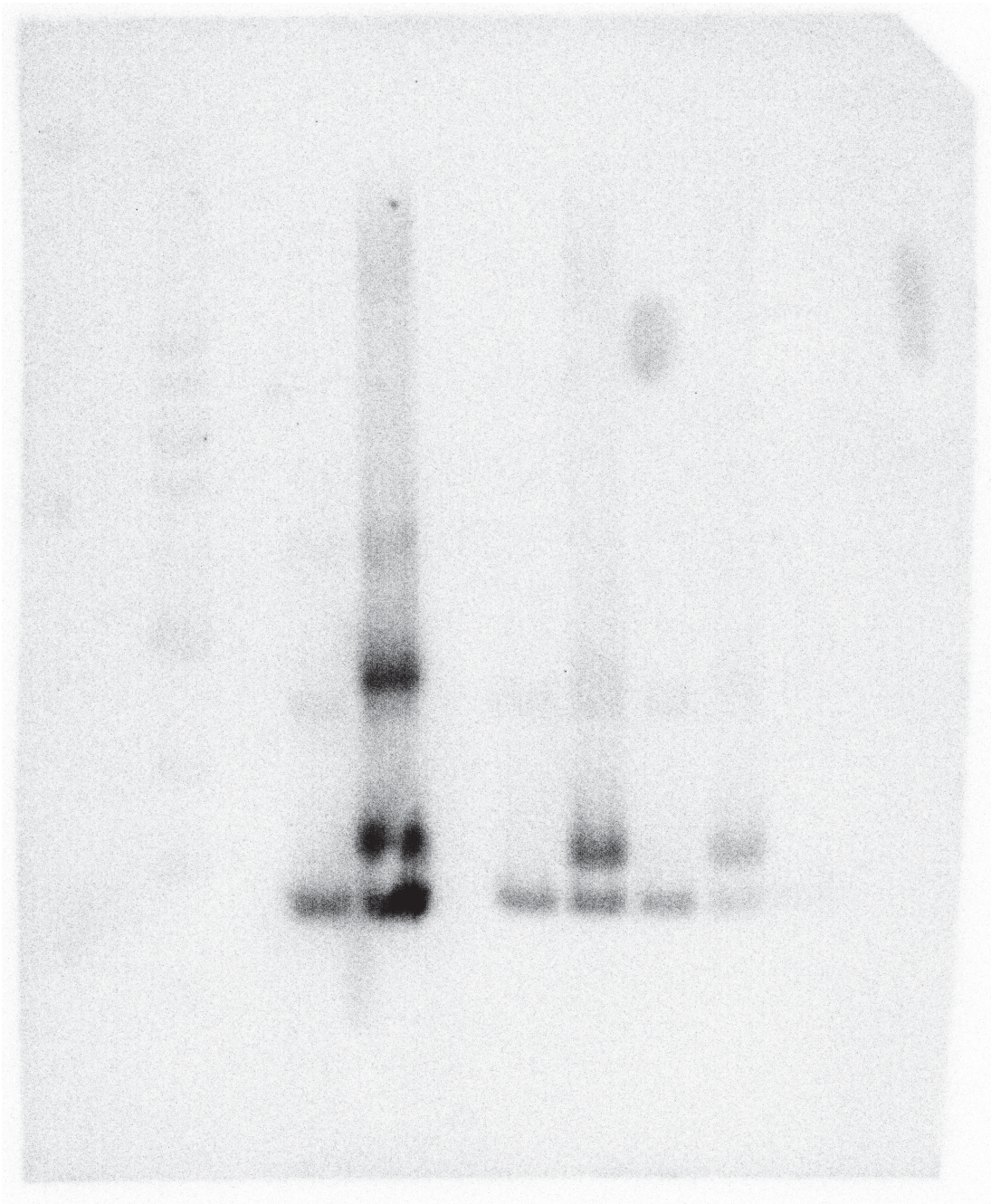

Supplement: Source Data Fig. 6 — Uncropped phosphor imager scan for Fig. 6. [file 41594_2023_956_MOESM9_ESM.pdf]

ED Fig. 2A

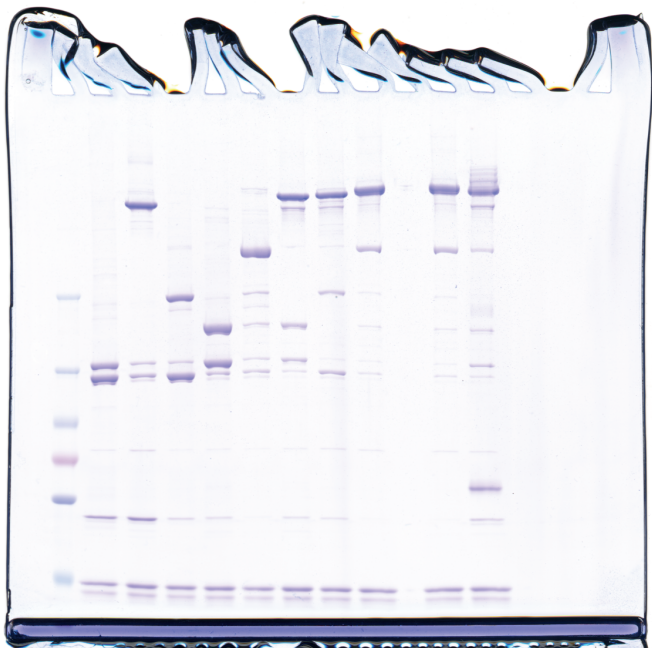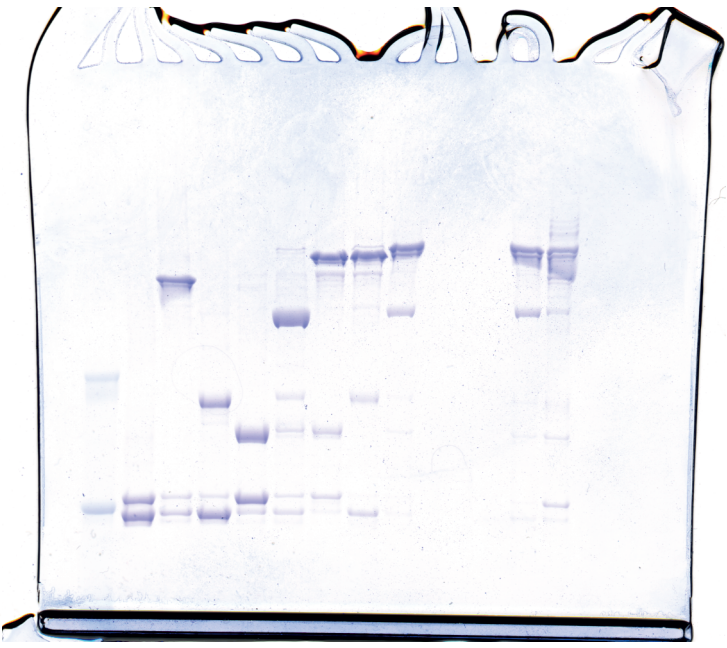

ED Fig. 2C

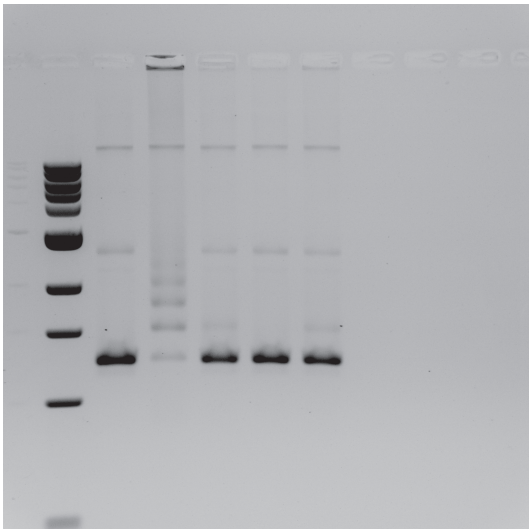

ED Fig. 2D

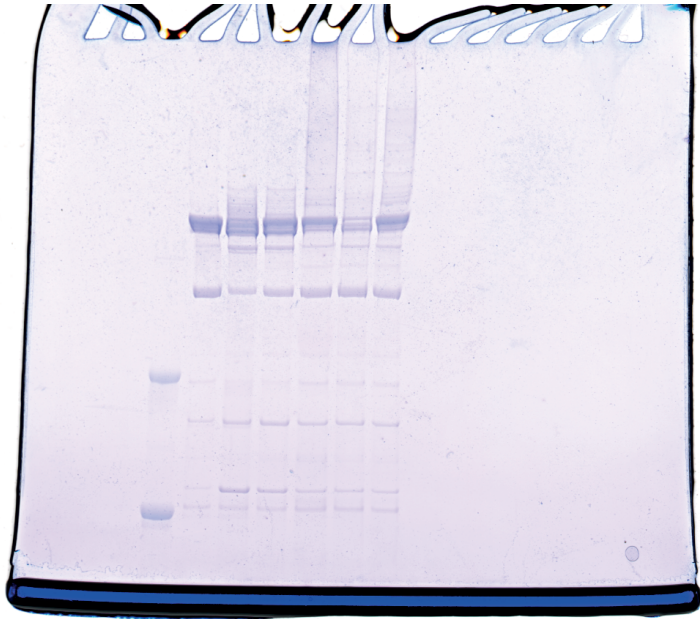

ED Fig. 2E

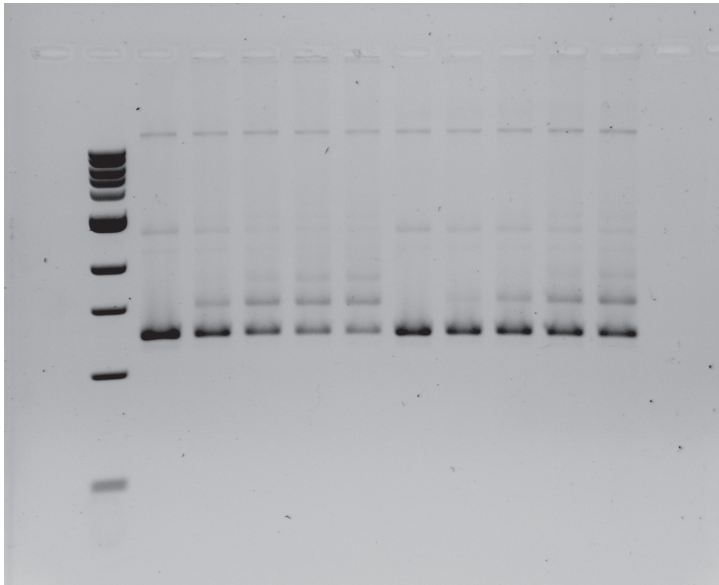

Supplement: Source Data Extended Data Fig. 2 — Uncropped pictures of agarose gels and protein gels for Extended Data Fig. 2. [file 41594_2023_956_MOESM10_ESM.pdf]

ED Fig. 4A

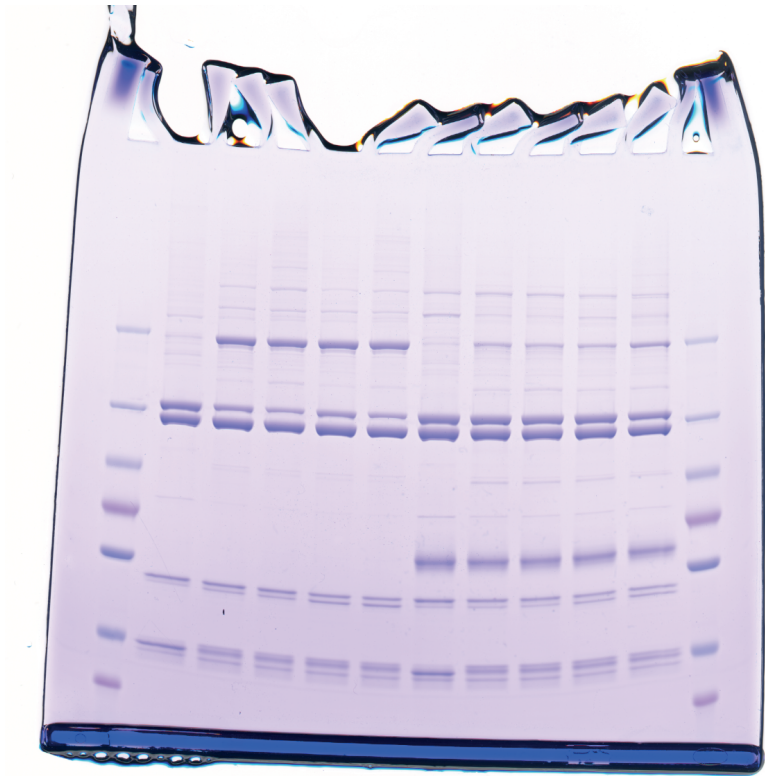

ED Fig. 4B

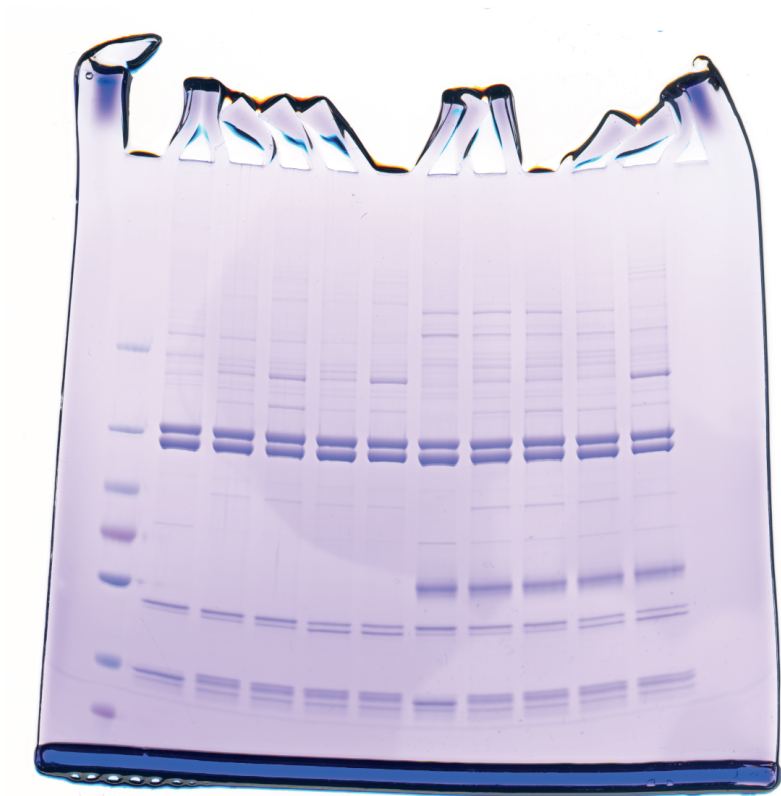

Supplement: Source Data Extended Data Fig. 4 — Uncropped SDS–PAGE pictures for Extended Data Fig. 4. [file 41594_2023_956_MOESM11_ESM.pdf]

ED Fig. 5B

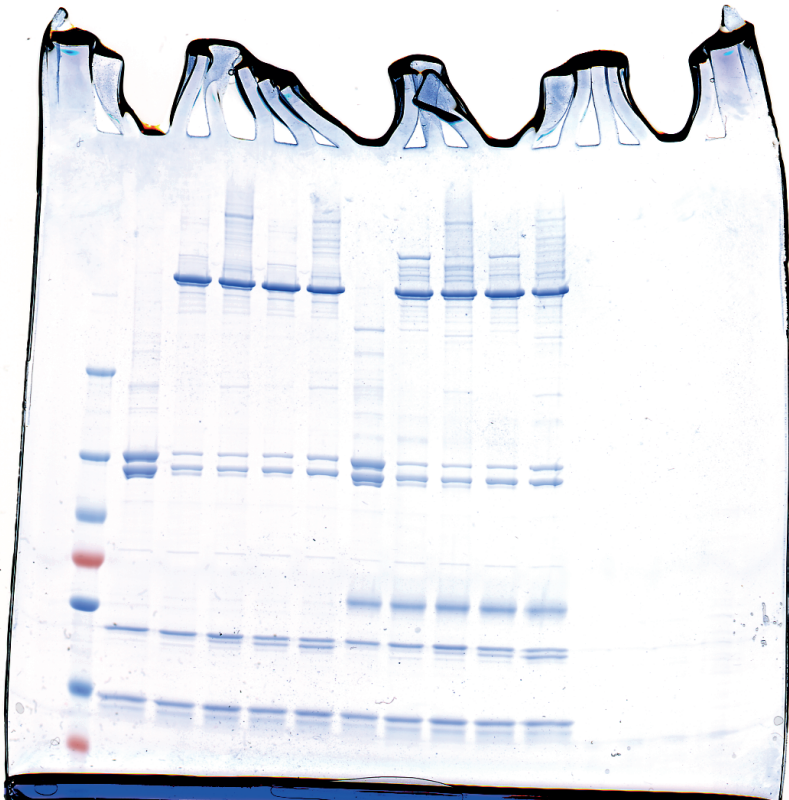

ED Fig. 5C

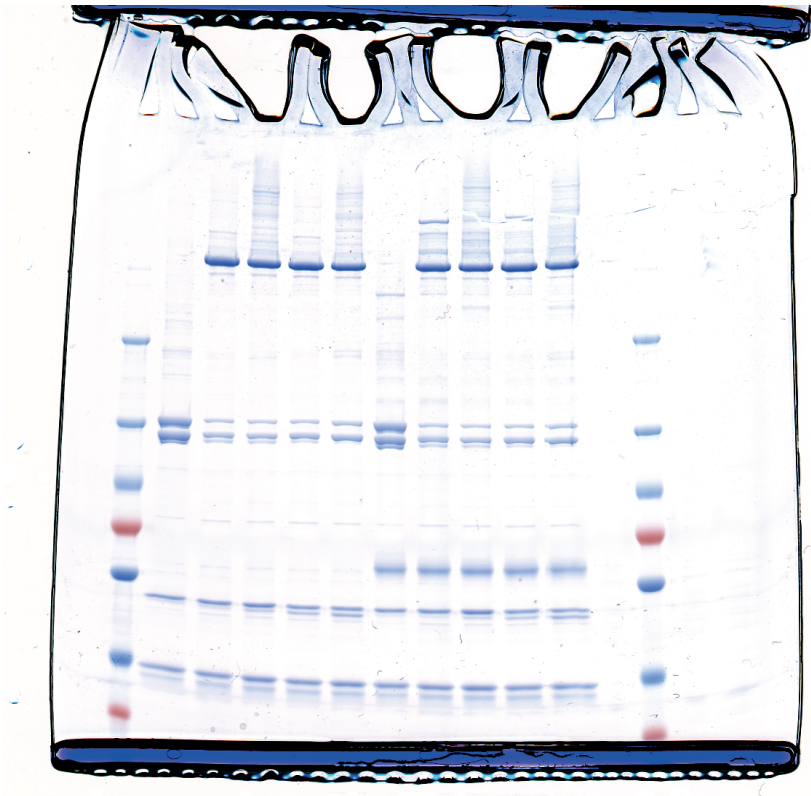

Supplement: Source Data Extended Data Fig. 5 — Uncropped SDS–PAGE pictures for Extended Data Fig. 5. [file 41594_2023_956_MOESM12_ESM.pdf]

ED Fig. 6A

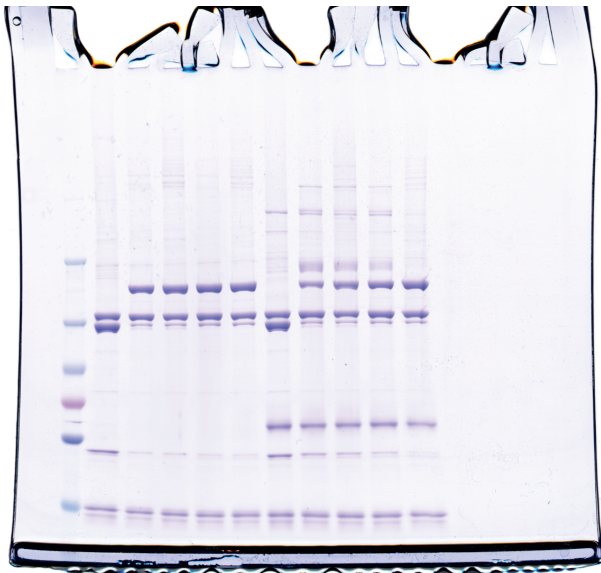

ED Fig. 6B

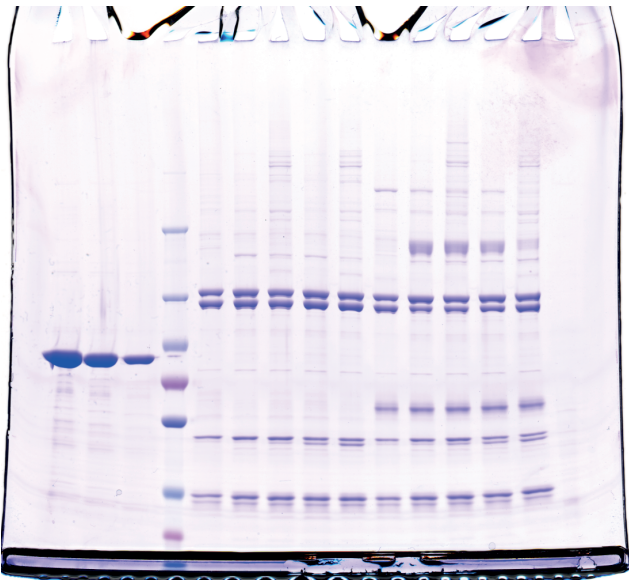

ED Fig. 6C

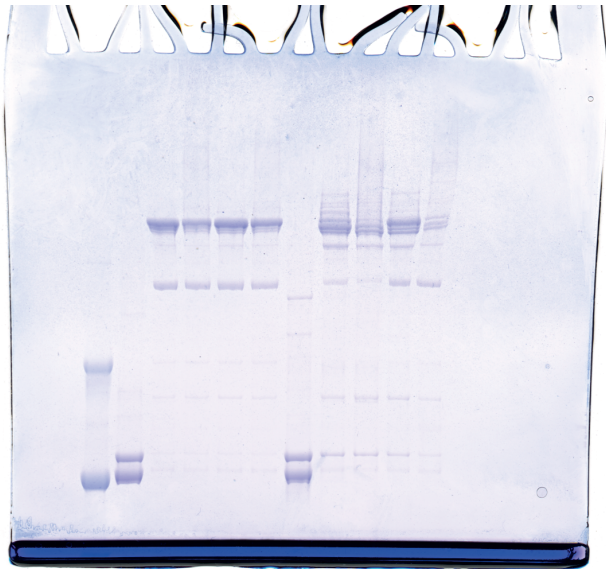

ED Fig. 6D

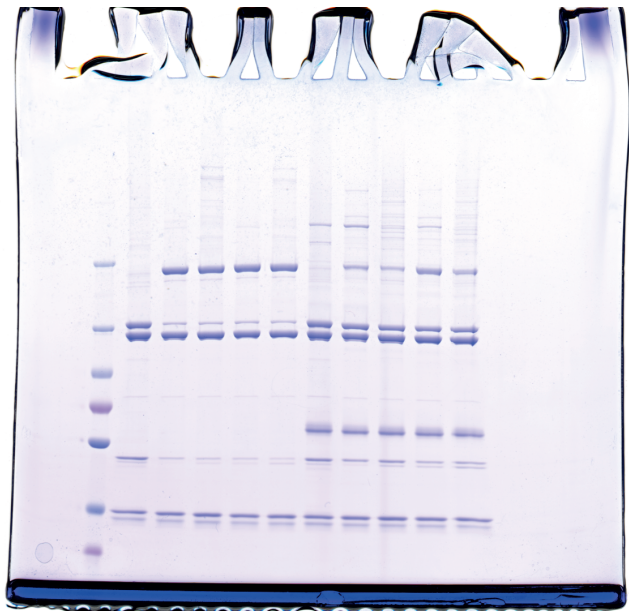

ED Fig. 6E

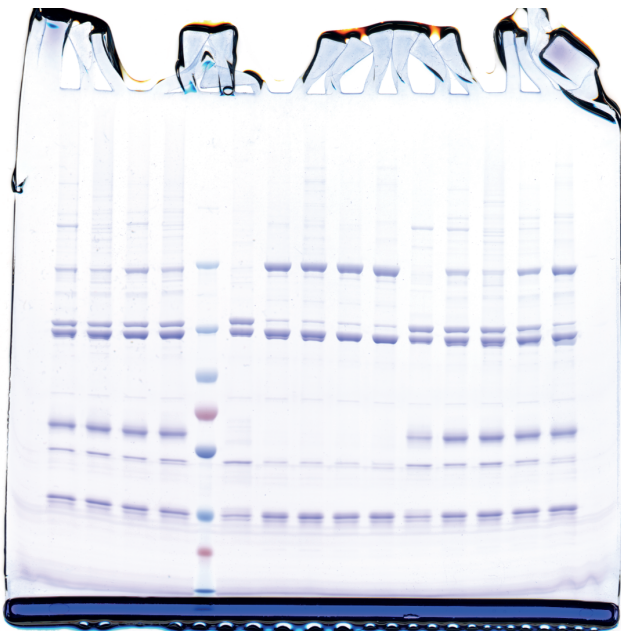

ED Fig. 6F

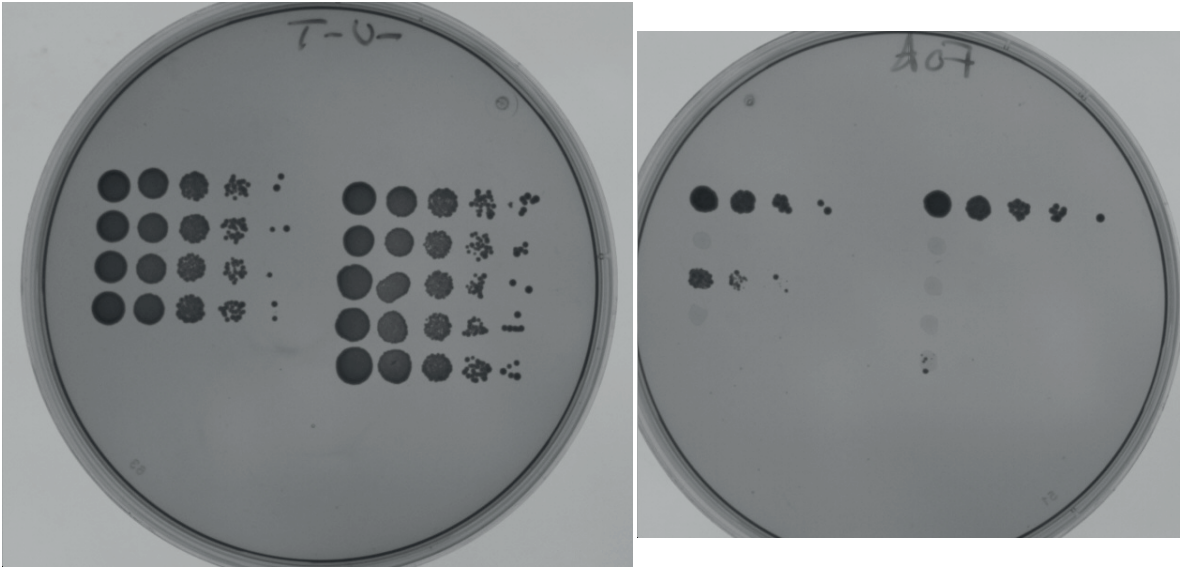

ED Fig. 6G

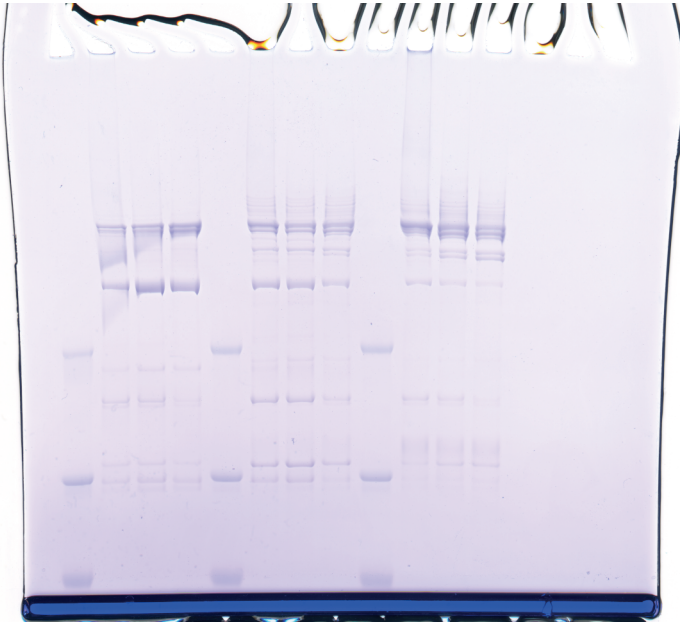

ED Fig. 6H

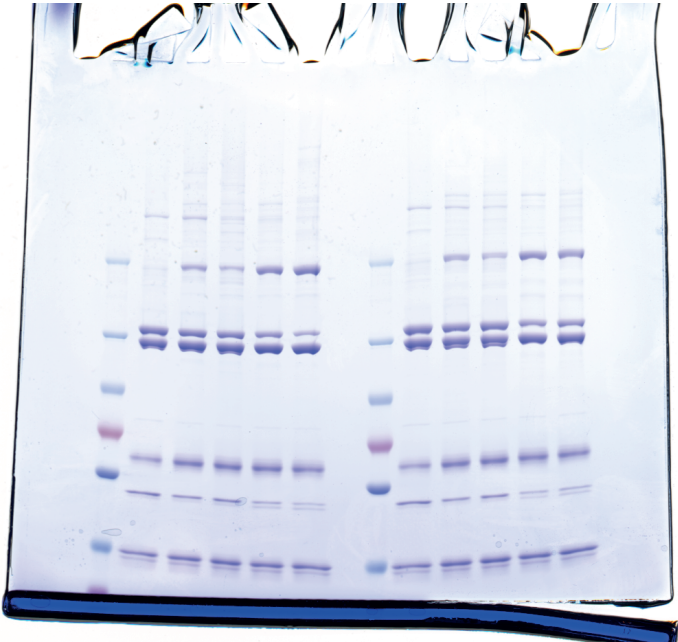

Supplement: Source Data Extended Data Fig. 6 — Uncropped SDS–PAGE and yeast spotting plate pictures for Extended Data Fig. 6. [file 41594_2023_956_MOESM13_ESM.pdf]

ED Fig. 7D and E

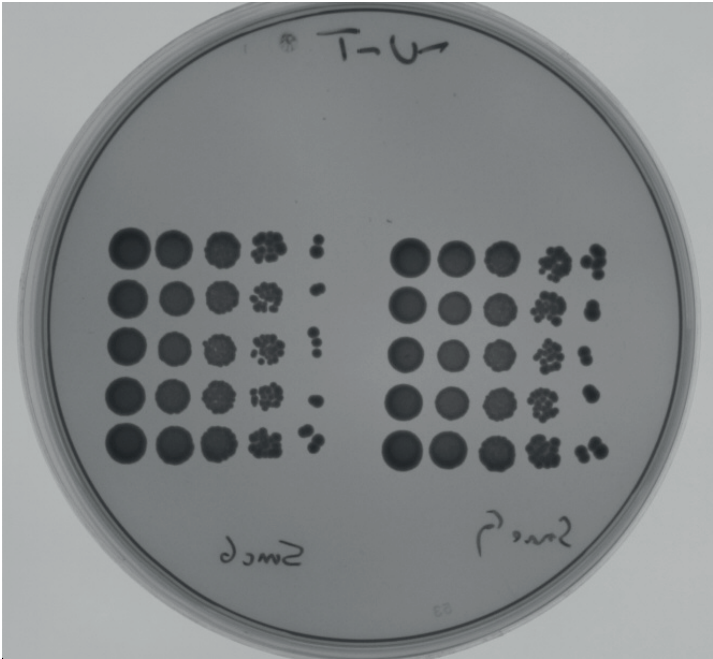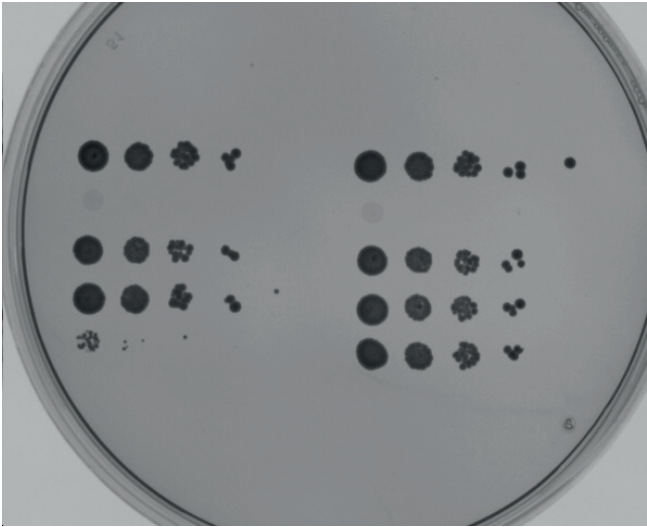

ED Fig. 7F

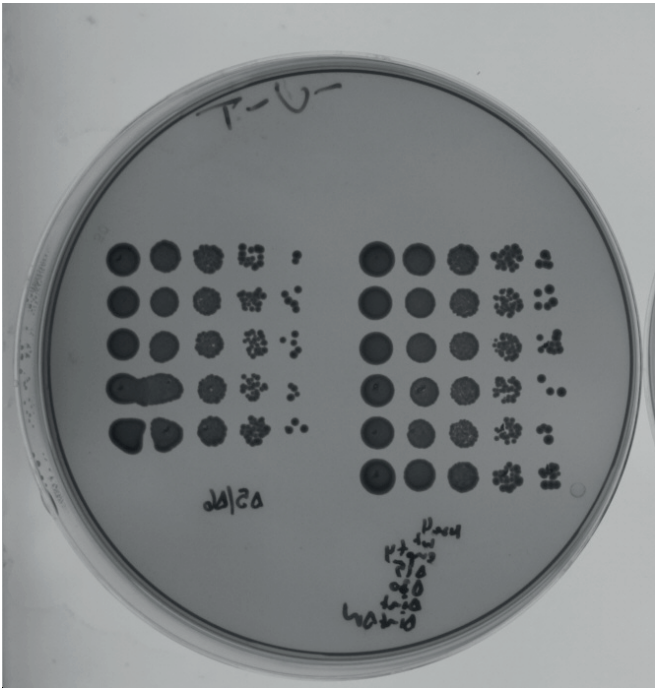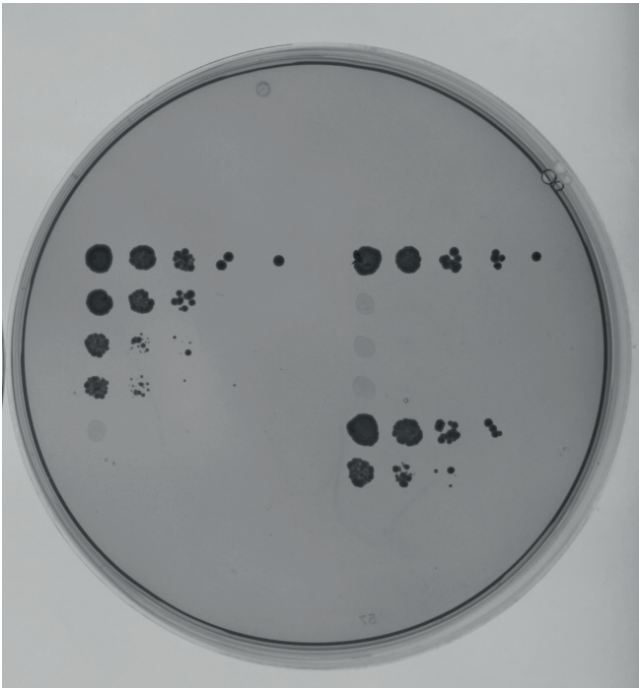

Supplement: Source Data Extended Data Fig. 7 — Uncropped yeast spotting plate pictures for Extended Data Fig. 7. [file 41594_2023_956_MOESM14_ESM.pdf]

ED Fig. 8D

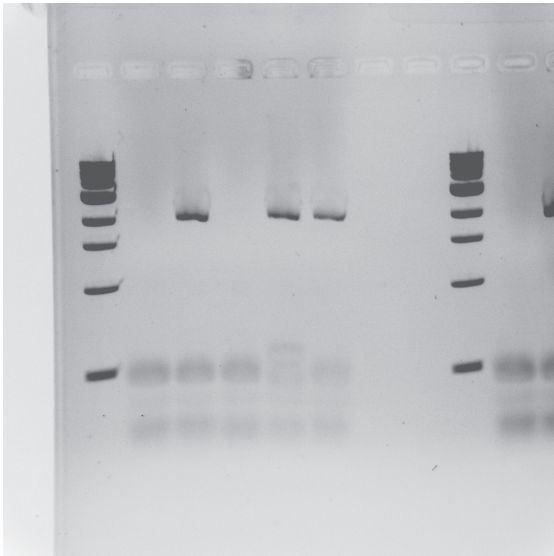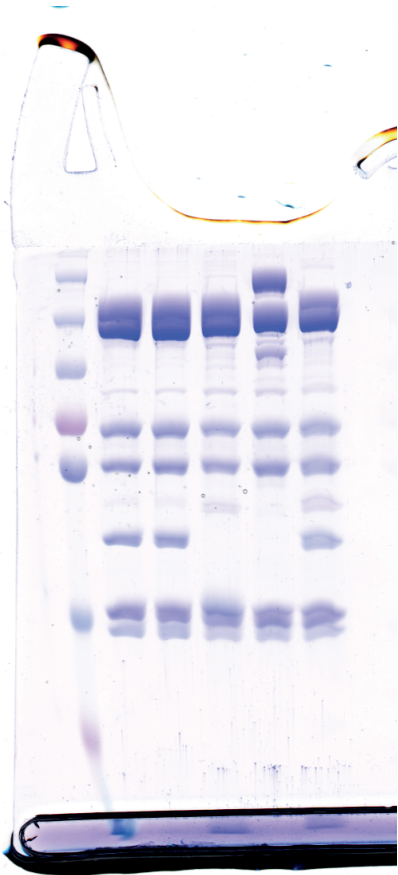

ED Fig. 8E

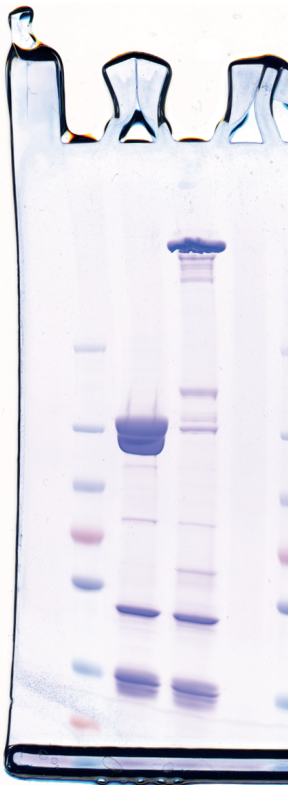

Supplement: Source Data Extended Data Fig. 8 — Uncropped pictures of agarose gels and protein gels for Extended Data Fig. 8. [file 41594_2023_956_MOESM15_ESM.pdf]
